# Boundary migration in a 3D deformed microstructure inside an opaque sample

**DOI:** 10.1038/s41598-017-04087-9

**Published:** 2017-06-30

**Authors:** Y. B. Zhang, J. D. Budai, J. Z. Tischler, W. Liu, R. Xu, E. R. Homer, A. Godfrey, D. Juul Jensen

**Affiliations:** 1 Section for Materials Science and Advanced Characterization, Department of Wind Energy, Technical University of Denmark, Risø Campus, Roskilde, 4000 Denmark; 2 Materials Science & Technology Division, Oak Ridge National Laboratory, P.O. Box 2008, Oak Ridge, Tennessee 37831-6064 USA; 3 Advanced Photon Source, Argonne National Laboratory, Argonne, Illinois 60439 USA; 4 Department of Mechanical Engineering, Brigham Young University, 435 CTB, Provo, UT 84602 USA; 5 Key Laboratory of Advanced Materials (MOE), School of Materials Science and Engineering, Tsinghua University, Beijing, 100084 P.R. China

## Abstract

How boundaries surrounding recrystallization grains migrate through the 3D network of dislocation boundaries in deformed crystalline materials is unknown and critical for the resulting recrystallized crystalline materials. Using X-ray Laue diffraction microscopy, we show for the first time the migration pattern of a typical recrystallization boundary through a well-characterized deformation matrix. The data provide a unique possibility to investigate effects of *both* boundary misorientation and plane normal on the migration, information which cannot be accessed with any other techniques. The results show that neither of these two parameters can explain the observed migration behavior. Instead we suggest that the subdivision of the deformed microstructure ahead of the boundary plays the dominant role. The present experimental observations challenge the assumptions of existing recrystallization theories, and set the stage for determination of mobilities of recrystallization boundaries.

## Introduction

Metals are irreplaceable high performance materials used widely in anything from the tiniest component to the largest construction in modern society. Processing of metals include conventional, yet often very advanced, thermomechanical treatments as well as a process which recently has become very popular, namely additive manufacturing by 3D printing^1^. Irrespective of the processing method, the properties of metallic materials depend largely on their microstructures, which can be controlled by thermal-mechanical treatments, i.e. by deformation and annealing. When metals and alloys are deformed, the energy stored in the materials is present as excess line and point defects, in the form of dislocations and vacancies. Upon annealing, the density of such defects is reduced through a number of mechanisms, among which recrystallization dominates and is the most important.

During recrystallization, new, almost defect-free, nuclei develop within the deformed matrix and grow by means of transfer of atoms from lattice arrangements in the deformed matrix into that of the growing nuclei, leading to the migration of the boundaries enclosing the nuclei through the deformation microstructure. The parameters generally assumed to be important for migration of recrystallization boundaries are the driving force for migration, based on the stored energy of the deformed microstructure, and the boundary mobility, which depends on crystallographic misorientation (determined by the crystallographic orientation of the crystals on either side of the boundary at each location) and the grain boundary plane^2–5^. Recently, it has been shown that curvature-based driving forces may also be important in determining the local migration characteristics. In the case of curvature-driven migration, the boundary stiffness also plays a role^6, 7^.

Using the differential aperture X-ray microscopy (DAXM) (also referred to as X-ray Laue diffraction microscopy) method^8–10^, which uses polychromatic synchrotron X-rays, it is possible now to characterize experimentally deformed microstructures in 3D non-destructively with sufficient spatial resolution to resolve the key length scale of the microstructure. In the present work, we use this technique to investigate local migration in 3D of a recrystallization boundary into a well-characterized deformed microstructure. Because the measurements are non-destructive, dynamic information about the motion of the recrystallization boundary can be obtained by a series of *ex-situ* annealing treatments. From these data sets we are able for the first time to investigate in full 3D the effects of the local deformed microstructure on migration of a recrystallization boundary.

An investigation of this type is not possible today with any other technique. *In-situ* electron microscopy techniques only reveal the microstructure in 2D, and the free sample surface(s) are likely to affect the results. Other synchrotron techniques such as 3D X-ray diffraction^11, 12^ and topo-tomography^13^ have insufficient spatial resolution to map the deformation microstructure in enough detail for such an investigation. Theoretical modeling using simulation techniques such as cellular automata^14^, Monte Carlo^15, 16^ and phase field models^17, 18^ generally use simplified conditions, and suffer from a lack of validated key input data, such as grain boundary energies, and in particular, grain boundary mobilities during the process of recrystallization.

## Results and Discussion

**Figure 1:**
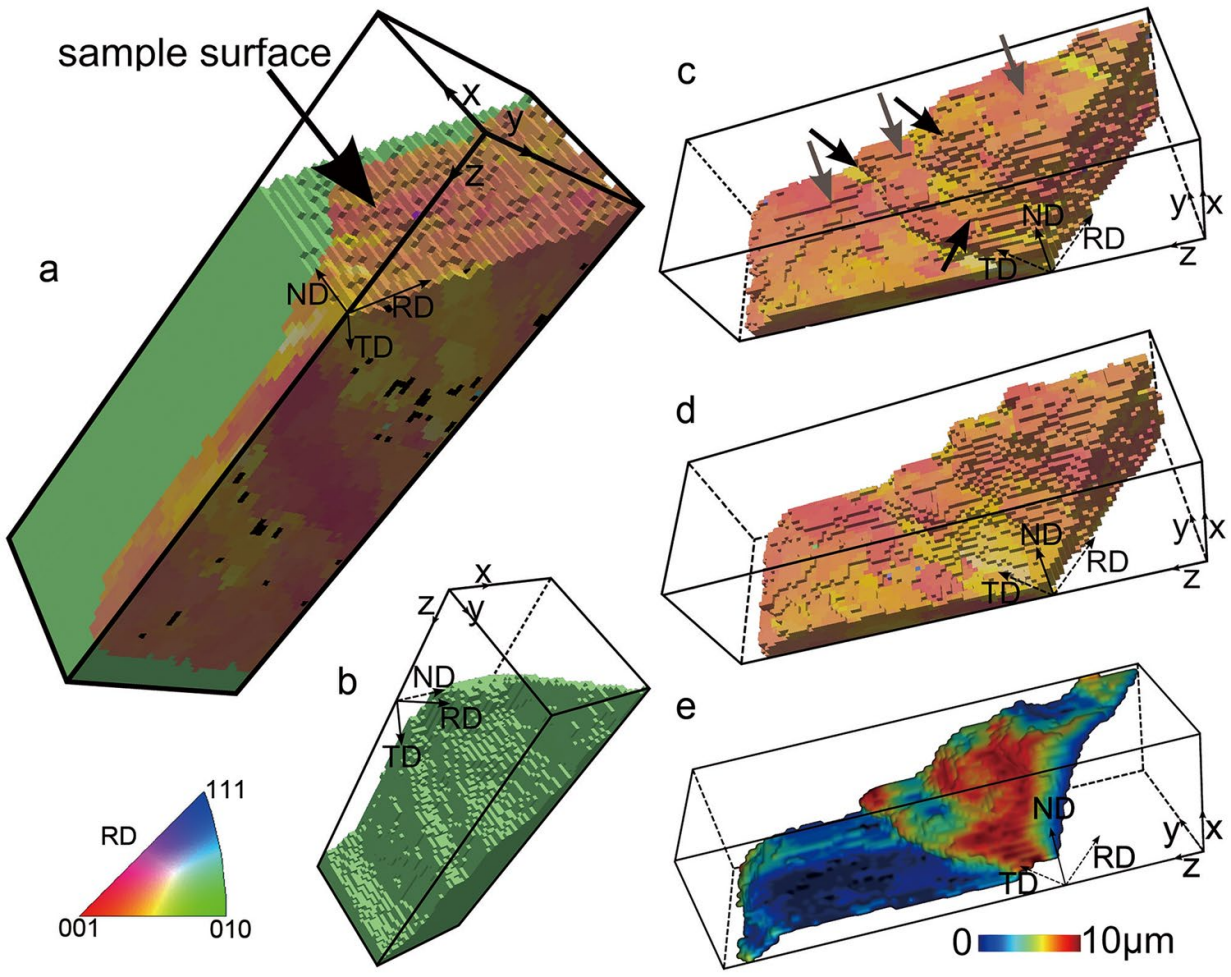
3D view of the gauge volume mapped by DAXM. (**a**) the whole volume mapped in the initial stage; (**b**) and (**c**) subsets of the volume shown in (**a**) visualizing the recrystallized grain and the deformed matrix, respectively. (**d**) the deformed part of the gauge volume after two annealing steps. The colors in (**a**)–(**d**) represent crystallographic orientations along the sample rolling direction (RD) (see the color code below (**a**)). (**e**) the initial recrystallizing boundary, showing how far each part migrates after the second annealing step (i.e. a plot of the migration distance). The color scale below the figure shows the magnitude of the migration distance. The black voxels in (**a**) represent non-indexed voxels. The dimensions along x, y, z, is 28, 32, 95 µm, respectively. The gray and black arrows in (**c**) mark faceted and curved parts of the boundary, respectively.

For the experiment, we used a high-purity partially recrystallized aluminum sample and followed the migration of a selected recrystallization boundary through its neighboring deformed material via a series of *ex-situ* annealing steps. The material was initially cold rolled to a reduction in thickness of 50%, and then annealed for 10 min at 250 °C to achieve a microstructure containing about 40% of recrystallized grains. A volume containing a selected typical high-angle recrystallization boundary was mapped with a step size of 1 µm using DAXM (see Fig. 1a). The boundary has a staircase shape consisting of flat parts (typically classified as facets^12, 13^, see Fig. 1c), which are approximately parallel to each other, and of curved parts connecting these facets.

**Figure 2:**
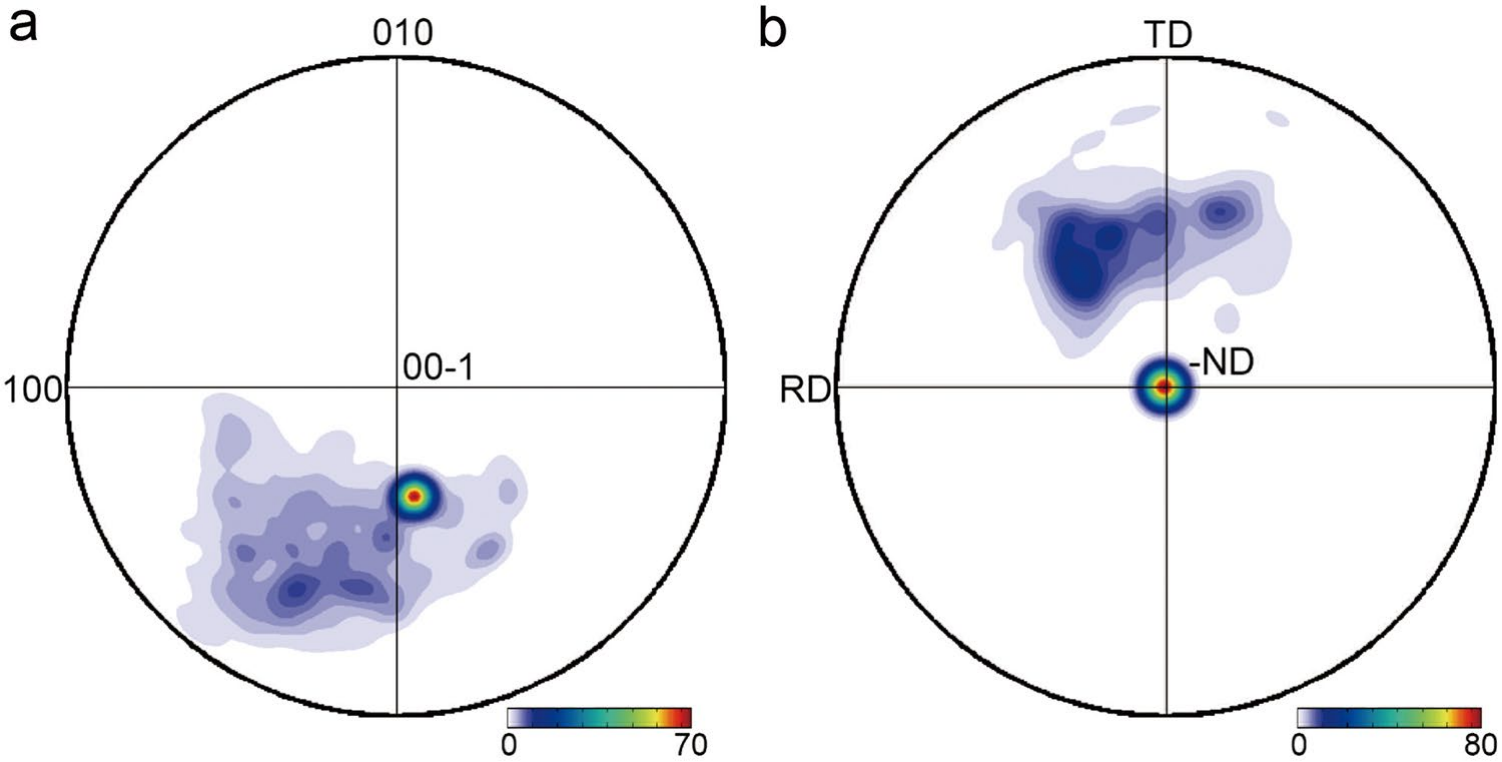
Distribution of boundary plane normals, determined at individual positions on boundary before annealing. The boundary plane normal is calculated in the crystal coordinate system of the recrystallized grain (**a**), and in the sample coordinate system (**b**). The strong peak in each figure corresponds to the facets observed in Fig. 1, while the clouds in the distribution are from the curved parts of the boundary.

DAXM directly gives local crystallographic data from which we can determine the full 5 parameters (3 for misorientation angles and 2 for boundary plane normals) that describe the recrystallization boundary. It is found that the plane normal of the faceted parts is close to [−0.05 −0.54 −0.83] in the face-centered cubic frame of the recrystallized grain, and is parallel to the sample normal direction (see Fig. 2). The average misorientation across the boundary is 53.6° <0.71 0.65 0.26>. Using this information, it can be determined that the facets are not coincident site lattice boundaries^19^, considered as special boundaries with a relatively low energy.

To follow the migration of the boundary into the deformed microstructure, the sample was annealed *ex-situ* at 260 °C in an air furnace, first for 17 min, and subsequently for another 30 min. After each annealing step, the sample was cooled in air, and then DAXM was used to map the same gauge volume as the initial mapped volume. In this way three positions of the boundary were obtained as it migrated.

During the two annealing steps, the boundary migrates, consuming parts of the deformed microstructure, resulting in growth of the recrystallizing grain. Figure 1d shows the remaining deformed part within the gauge volume after the second annealing step. By comparing this with Fig. 1c, the volume that has been consumed by the recrystallizing grain can be obtained. To better visualize the migration of the recrystallization boundary, the distance from each voxel on the initial boundary to the new boundary position after the second annealing step is calculated, determined as the minimum distance between a given voxel on the initial boundary and any voxel on the boundary after the annealing, and is defined as the migration distance. Figure 1e shows a color mapping of the migration distance as a function of the initial boundary position.

A key observation is that the facets do not migrate as much as the curved parts. Inspection of the data shows that the boundary migrates by movement of the curved parts laterally along the facets, thereby maintaining the staircase shape of the boundary. As a result, the facet edges are either extended or shortened during boundary migration. Interface migration by ledge movement is a well-established mechanism for the movement of interphase boundaries during phase transformations^20^, and a detailed atomistic model, in which boundary migration takes place by the movement of steps or kinks in the boundary, has been proposed^21^. It is interesting that the migration we observed of the recrystallization boundary resembles this ledge mechanism although the scales are totally different: in our case on micrometer scale, while for classic boundary migration the ledge movement is on the atomic scale.

As a result of the high spatial resolution and the non-destructive nature of the DAXM technique, we also have detailed information about the deformation microstructure consumed by the migrating recrystallization boundary, allowing the differences in migration rate to be further investigated. For this, the results are first analyzed based on the classic equation for boundary migration, where the boundary migration rate, v, can be expressed as^2^:

1 $${\rm{v}}=\text{MF},$$

where M and F are the boundary mobility and driving force, respectively.

Based on this equation, the observed differences in migration distance could arise if the driving force, F, in the deformed matrix in front of the facets is lower than that in front of the curved parts, and/or if the mobility, M, for the facets is lower than that for the curved parts. The mobility is generally assumed to depend on the boundary misorientation and the boundary plane normal^4, 5^. In the following, we therefore analyze if any of these three factors (driving force, boundary misorientation angle, and boundary plane normal) can explain the different migration of the facets and the curved boundary segments.

For the analysis, the recrystallization boundary is separated into migrating and non-migrating parts, by comparing the position of the recrystallization boundary between two successive annealing steps. The non-migrating parts are defined as those parts that migrate less than 1 µm (which is the resolution of the current DAXM mapping), with the remaining volume defined as the migrating parts. This partitioning shows that the migrating parts are mainly curved segments of the boundary, while the non-migrating parts are mainly the facets (see Supplementary Fig. S5). Additionally, the deformed volume that has been consumed by the recrystallized grain during a given annealing step can be determined as the volume between the two boundary positions before and after the annealing step.

To test the importance of the driving force, values of stored energy in the deformed matrix in front of the migrating and non-migrating parts have been estimated. The stored energy consists of contributions from the dislocation boundaries and incidental loose dislocations as well as from long range elastic strains. Therefore the stored energy is calculated by taking the product of the boundary energy per unit area (determined using the boundary misorientation angle according to the Read-Shockley equation)^22^ and the area per unit volume of the boundary (see supplementary text for details). The influence of short–range elastic stresses is included in the Read-Shockley equation (as a result of stress-screening of dislocations within the boundaries). The effects of longer range elastic stresses and loose dislocations are, however, not considered, as these cannot be determined from the present data, and in any case are not expected to differ greatly between the regions in front of the flat and curved parts of the boundary.

For the migrating parts, the stored energy is calculated directly from the consumed volume, while for the non-migrating parts, the stored energy is calculated taking a volume of 2 µm in thickness in front of the non-migrating boundary segments (the value of 2 µm is chosen to give a volume of similar size as for the migrating parts). It is found that for the first annealing interval the average stored energies for the migrating and non-migrating parts are 0.19 ± 0.02 MJ/m^3^ and 0.17 ± 0.01 MJ/m^3^, respectively. During the second annealing interval, the values are 0.21 ± 0.02 MJ/m^3^ and 0.17 ± 0.02 MJ/m^3^, respectively. Although the stored energy in front of the non-migrating parts of the boundary is ~20% lower than the stored energy of the deformed material consumed by the migrating parts, the difference in stored energy is not sufficient to explain why such a large fraction of the curved parts migrate as much as 8–10 µm (at rate of ~0.2 µm/min), while the facets migrate less than 1 µm (only ~20% difference would be expected according to equation (1)).

**Figure 3:**
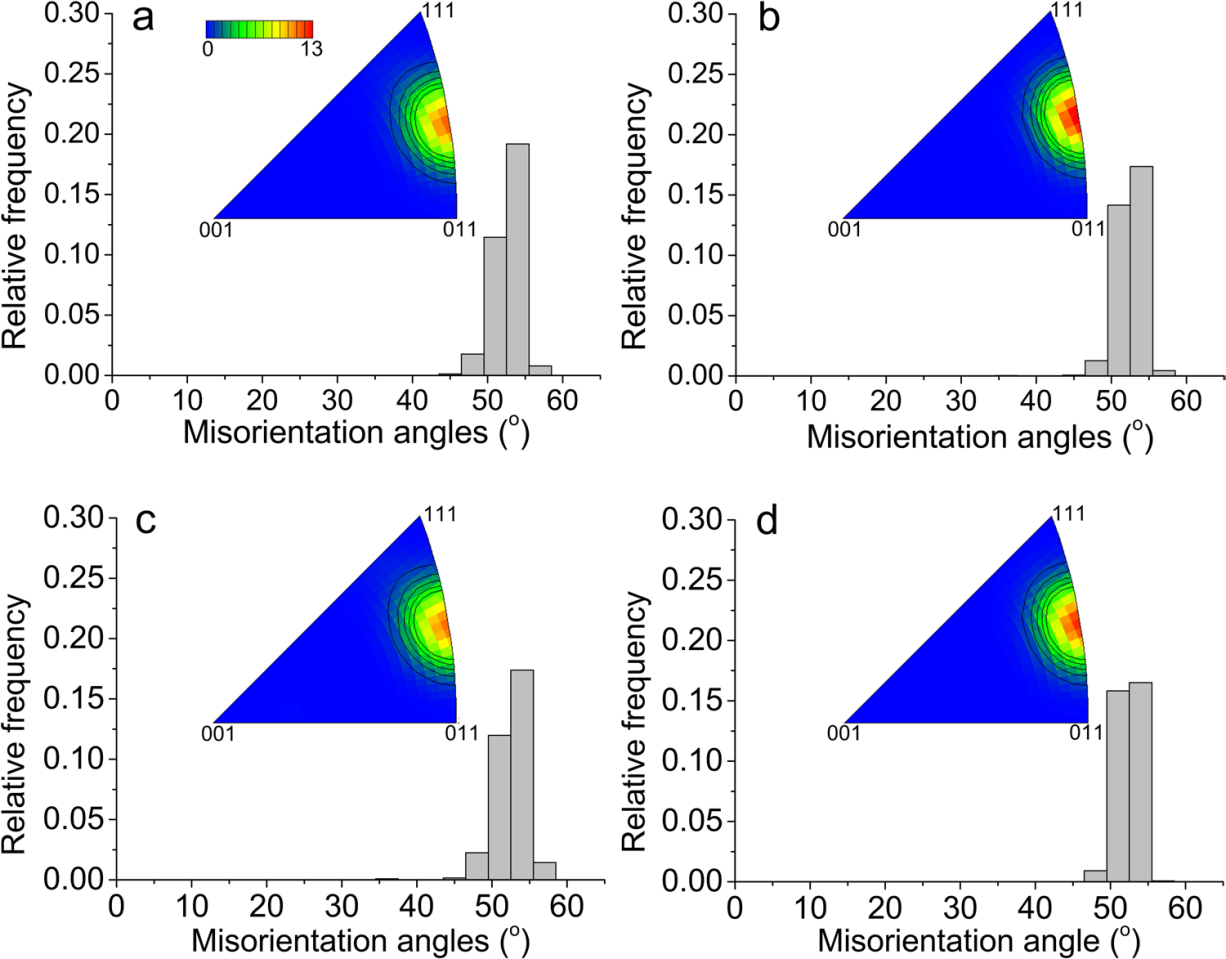
Misorientation distribution for the migrating and non-migrating boundary segments. (**a**,**b**) and (**c**,**d**) show data from the first and second annealing steps, respectively. (**a**) and (**c**) are for migrating parts, while (**b**) and (**d**) are for non-migrating parts of the boundary.

To test the importance of the boundary misorientation, the values across the two types of boundary (migrating and non-migrating) are calculated. The misorientations across the non-migrating boundary parts are directly calculated by taking the misorientation between all sets of two neighboring voxels across each location on the boundary. For the migrating parts, it is necessary to consider the misorientations between the recrystallized grain and all voxels in the deformed volume consumed during annealing, as all these voxels are, or were neighbors to the migrating boundary at some stage during annealing. It is found that misorientations for the migrating and non-migrating segments are quite similar (see Fig. 3). Therefore we conclude that the misorientation cannot be the reason for the observed migration differences.

To test the importance of boundary plane normal, values for the facets and for the curved parts are compared. As shown in Fig. 2 it is evident that the distributions for migrating curved parts and non-migrating facets differ significantly, suggesting that the grain boundary plane normal could be the reason for the observed variation in migration behavior of different parts of the recrystallization boundary.

The most direct way to test this hypothesis further would be to determine values for mobilities directly from the present experimental data. For the curved boundary parts this could indeed be done. However, as the facets do not migrate during either of the two annealing steps, values for mobility cannot be determined (or are trivially equal to zero), and such a calculation does not therefore help the present analysis. Alternatively, molecular dynamics (MD) simulations can be used to simulate the migration of specific boundaries in bicrystals under various driving forces^6, 23, 24^ to evaluate the differences in boundary mobility. Recently, MD simulations have been conducted to predict boundary mobilities for three boundaries with fixed misorientation (similar to that of the present boundary) but with different boundary plane normals under a range of driving forces^25^. One of the boundary plane normals is the same as that found for the experimental facets, while the other two normals are within the observed cloud for the curved parts in Fig. 2. The MD simulations predict mobilities of the same order for all three boundaries, with even larger mobilities predicted under some conditions for the boundary with normal identical to that of the immobile facets^25^. Similar conclusions have been reached for Σ5 boundaries with different boundary plane normals^7^.

One may speculate whether within the cloud of grain boundary normals for the curved parts there may nevertheless be boundary planes that have higher mobilities than the chosen two cases, and that these mobilities may be much higher than the value calculated for the facets. Although this is possible, it has to be considered that *all* the curved boundary parts, covering a cloud of grain boundary plane normals, are observed experimentally to migrate faster than the facets. Thus it seems more likely that it is the curved nature of the boundaries and the local influence of the deformation microstructure, rather than a grain boundary plane normal dependence, that leads to the faster migration of curved parts of the boundary than of the facets.

Recently, quantitative analysis of curvature of recrystallization boundaries has shown that locally the boundary curvature can provide driving or dragging forces comparable to that of the stored energy^26, 27^. For the present boundary, this is the case only for the sharp corner marked by the black arrow to the left in Fig. 1c, where a minimum 3D mean curvature radius is about 3–5 µm, corresponding to a driving force of 0.1–0.2 MJ/m^3^. The majority of the boundary segments have mean curvature radii in the range 30–100 µm, providing driving forces below 0.02 MJ/m^3^. More importantly, the curved parts connected to the right side of the largest facets migrate against the curvature. Therefore, we believe the local curvature-based driving force is not the main reason for the present boundary.

**Figure 4:**
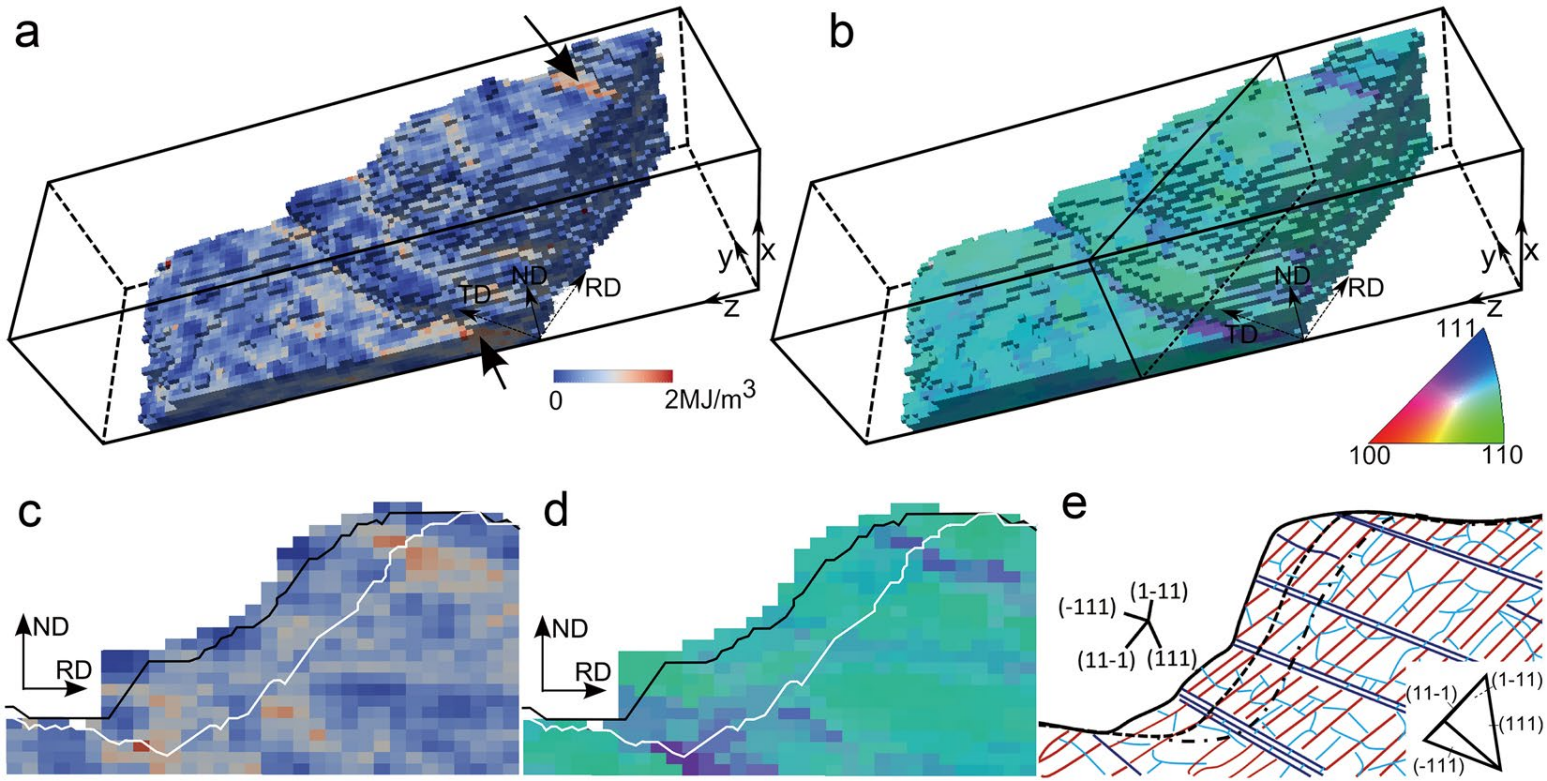
Effects of the deformation microstructure on the local boundary migration. The deformation matrix at the initial stage colored according to the local stored energy (**a**) and local misorientation rotation axis (**b**). (**c**) and (**d**) show a cross-section view of the microstructure at the position marked in (**b**). Black and white lines mark the boundary positions after the two annealing steps. (**e**) Sketch drawn based on the 3D X-ray data and 2D data from electron microscopy showing the microstructure and the boundary traces. The blue and red lines represent the two sets of intersecting dislocation boundaries, while the light blue lines represent incidental dislocation boundaries, all of which are typically observed in deformed aluminium at strains similar to that of the present sample^28^. The black solid, dashed and dot dashed lines represent the three recrystallization boundary positions. The traces of the four {111} planes and the projection of a {111} tetrahedron for the deformed grain on the RD-ND section are also shown.

The present experimental data allow further analysis of such possible effects of the local deformed microstructure ahead of the recrystallizing boundary. For better visualization, the deformation matrix shown in Fig. 1c is re-colored according to the local stored energy and local misorientation rotation axis in Fig. 4a and b, respectively. For both the facets and the curved boundary parts, a mix of colors is seen in Fig. 4a and b, which is a result of local orientation variations in the deformed microstructure. Some of the volumes with high local stored energies, marked by arrows in Fig. 4a, are aligned in bands. Such bands are commonly found in cold rolled metals^28^.

To correlate the deformed microstructure with the boundary migration, a typical section along the rolling direction (RD) – normal direction (ND) plane at the position marked in Fig. 4b is shown in Fig. 4c and d, where the boundary positions after the two annealing steps are shown by lines. The deformed matrix in the section is seen to be subdivided by two sets of intersecting boundaries inclined about 40° and −20° to RD, which are aligned nearly parallel to the traces of the (11–1) and (−111) planes, respectively (shown by the sets of blue and red lines in Fig. 4e). The set that is parallel to (−111) has generally higher misorientations, which therefore results in large color variations at the corresponding locations in both the stored energy and misorientation maps (see Fig. 4c,d). From inspection of the boundary position at each annealing step, it is evident that the migration of the curved parts follows the (−111) set of dislocation boundaries. The migration of the curved part is likely to be promoted by these favorably aligned dislocation boundaries.

This observation supports a previous suggestion based on destructive 3D investigations^29^, where no dynamic (*in-situ*) data were available. The influence of the local arrangement of the dislocation boundaries in the deformed microstructures on the migration of recrystallization boundaries has also been observed on 2D sample surfaces^26, 27, 30^. With the present 4D data sets, we are able for the first time to reveal the influence of the 3D arrangement of the local deformed microstructure on the complex migration pattern of a recrystallization boundary in the bulk.

## Conclusions

In the present study, DAXM has been used to characterize boundary migration during recrystallization. A typical high angle recrystallization boundary (of non-special character) has been followed as it migrates through the deformed matrix of pure aluminum cold rolled to 50%. It was found that on a local scale the investigated boundary formed extended planar facets, with plane normals parallel to the sample normal direction, and with the facets connected by curved parts of the boundary. A key observation is that the curved parts of the boundary migrate more quickly than the faceted parts. In analyzing the reasons for this difference, it is shown that neither differences in local average driving force nor differences in misorientation can explain the results. Moreover, although a systematic difference in boundary plane is found for the faceted and the curved parts of the boundary, by combining the experimental results with MD simulations we conclude that this also is not the major reason for the difference in boundary migration. Instead it is suggested that boundary migration during recrystallization is strongly affected by the local geometrical arrangement of the dislocation boundaries in the deformed microstructure.

As described here, DAXM offers the fascinating possibility to measure both the boundary migration velocity and the driving force, as determined from the energy stored locally in the deformed matrix in front of the migrating boundary. It remains however as an open question how the mobility of a recrystallization boundary, which changes significantly in space and in time, should be considered. DAXM provides, nevertheless, a unique opportunity for addressing this question as it allows small parts of a migrating 3D boundary, which will typically have a range of different misorientations and boundary plane normal (see Fig. 4c and d), to be analyzed separately, and hence to allow the variation in local boundary mobility to be determined.

## Methods

### Sample

A polycrystalline pure aluminum (99.996%) with large initial grain size of several millimeters was used as a starting material. The high purity was chosen to reduce possible effects of particles or impurities, and a polycrystalline grain structure was chosen to ensure the development of more general types of recrystallization boundaries, rather than the special boundaries (e.g. 40° <111> boundaries) that are often seen in growth selection experiments during recrystallization of deformed single crystals^3^. The sample was rolled to a 50% reduction in thickness and then annealed at 250 °C for 15 min to start the recrystallization process. 27 recrystallization boundaries on the longitudinal section (defined by the normal direction (ND) and rolling direction (RD) of the sample) were characterized using electron channeling contrast (ECC) and an electron backscattering diffraction (EBSD) system attached to a Zeiss 35 scanning electron microscope. The sample was then further annealed *ex situ* at 250 °C for another 15 min and 30 min to observe the progress of recrystallization on the surface of the sample. It was found that among the 27 boundaries, 22 migrated in stop-go fashion, 3 migrated all the time and 2 did not migrate during the annealing periods. Based on these observations, one boundary migrating in stop-go fashion was chosen for the 3D study. This stop-go migration is representative of a general migration pattern for recrystallization boundaries that has been seen in many in-situ or *ex-situ* 2D^26, 27, 30^ and 3D studies^12, 13^. To further ensure success of the synchrotron experiments, the sample was annealed at 260 °C for another 10 min and 30 min to confirm the migration behavior of this selected boundary. A length of recrystallization boundary consisting of both curved and flat segments after annealing, where the boundary was observed to migrate at a relatively predictable speed was selected as candidate region for the 3D measurements (see Supplementary Fig. S1).

### Synchrotron experiment

The micro-beam X-ray Laue Diffraction Microscope (or Differential Aperture X-ray Microscope, DAXM)^31^ at beam line 34-ID-E at the Advanced Photo Source (APS) in Argonne National Laboratory was used to map the 3D volume containing the area marked by the rectangle in Supplementary Fig. S1. A schematic of the experimental setup is shown in Supplementary Fig. S2. In the DAXM experiment, a polychromatic X-ray beam, with energies in the range of 7–30 keV, was focused using two non-dispersive Kirkpatrick-Baez focusing mirrors, producing a beam with a Lorentzian profile with a full width half maximum of ~0.5 µm. The sample was mounted on an inclined sample holder at a 45° incidence angle to the X-ray beam, and was scanned horizontally and vertically by moving the sample stage. At each position of the focused beam on the sample surface a Pt-wire of 50 µm in diameter was used as a differential aperture, which was scanned continually (in so-called fly-scan mode) in a plane parallel, to the sample surface at a distance of ~100 µm. The scanning range of the wire was optimized to cover all diffracted beams coming from a volume illuminated by the beam down to ~100 µm below the sample surface. Such wire scans were carried out at each position of the beam in a grid of size 28 × 32 with grid spacing of 1 µm × 1 µm.

During each wire scan, the Laue diffraction pattern from all grains intercepted by the incident beam at each scanned position was recorded on a Perkin-Elmer flat panel detector (409.6 × 409.6 mm^2^, 2048 × 2048 pixels, amorphous Si, CsI scintillator, 16-bit dynamic range corresponding to 65536 counts) mounted in 90° reflection geometry 510.3 mm above the sample. The origin of the scattered contributions arising from different depths along the beam was determined by ray-tracing from the wire scan patterns, allowing the reconstruction of depth-resolved Laue patterns^32^. Reconstructions were carried out to a depth of ~100 µm into the sample, also with a step size of 1 µm. For each depth-reconstructed pattern, orientation indexing of the diffraction patterns was performed using the LaueGo software at 34-ID-E beamline. As a result a mapped volume of size of 28 × 32 × 95 µm^3^, with a 1 µm^3^ voxel size, was obtained (see Fig. 1). The 3D volume was analyzed using Matlab and visualized using Dream3D/Paraview^33^.

## Electronic supplementary material

Supplementary text
