# Supplementary material for: Boundary migration in a 3D deformed microstructure inside an opaque sample
Source: Sci Rep. 2017 Jun 30;7:4423. doi: 10.1038/s41598-017-04087-9 (PMC5493684; doi:10.1038/s41598-017-04087-9)
Supplement: Supplementary file 1 — Supplementary text [file 41598_2017_4087_MOESM1_ESM.pdf]

Supplementary Materials

Boundary migration in a 3D deformed microstructure inside an opaque sample

Y. B. Zhang [1*], J. D. Budai [2], J. Z. Tischler [3], W. Liu [3], R. Xu [3], E. R. Homer [4], A. Godfrey [5], D. Juul Jensen [1]

[1] Section for Materials Science and Advanced Characterization, Department of Wind Energy, Technical University of Denmark, Risø Campus, Roskilde 4000, Denmark

[2] Materials Science & Technology Division, Oak Ridge National Laboratory, P.O. Box 2008, Oak Ridge, Tennessee 37831-6064, USA

[3] Advanced Photon Source, Argonne National Laboratory, Argonne, Illinois 60439, USA

[4] Department of Mechanical Engineering, Brigham Young University, 435 CTB, Provo. UT 84602, USA

[5] Key Laboratory of Advanced Materials (MOE), School of Materials Science and Engineering, Tsinghua University, Beijing 100084, P.R. China

*Correspondence to: yubz@dtu.dk

Sample alignment For ex-situ annealing, it was necessary to remove the sample from the holder and then remount it after each annealing treatment. To ensure alignment of the sample after each annealing step, a fiducial Pt cross was deposited on the electropolished sample surface close to the volume chosen for investigation (Fig. S3a). Before each mapping, the area around fiducial Pt mark was scanned using a fluorescence detector to find the center of the cross. Using this method the accuracy of determination of the center coordinate is better than 1µm along both the x- and y-directions (Fig. S3b). Additionally, the sample was fixed mechanically on a specially designed holder, in order to further minimize alignment errors during remounting sample after each annealing step, in particular to reduce the sample rotation. The measured orientation of the recrystallized grain before and after the annealing steps differed by only a misorientation angle of ~0.3° (note that the angular resolution of the present technique is better than ~0.1°), which implies that largest misalignments within the volume are less than 1µm. Considering the alignment error from both the translation and rotation, the accuracy of the alignment is estimated at approx. ±1µm along all three directions.

Boundary plane normal In order to obtain the boundary plane normal information, the extracted voxel-based image of the recrystallizing boundary was first smoothed twice with 3x3 Gaussian filter, as illustrated in Fig. S4s and b, showing the raw data and smoothed boundary before annealing, respectively.

After smoothing, the boundary was then triangulated using the built-in Matlab 'isosurface' function. The surface normal for each triangle on the boundary represents the normal at that position on the boundary. The distribution of boundary plane normals was then obtained by plotting all vectors of the triangle normals in standard stereographic projection using the MTEX software package [1]. The result for the recrystallization boundary before annealing is shown in Fig. 2.

Partitioning into migrating and non-migrating boundaries By comparing the boundary position at different annealing steps, the migration of the boundary was quantified. In the present work, the minimum 3-D Euclidean distance from each voxel on the initial boundary to the new boundary position after annealing was calculated and defined as migration distance. Considering the estimated accuracy in alignment for the present study, boundary voxels that migrate less than 1 pixel are classified as non-migrating, with the remaining boundary voxels classified as migrating segments. The result of this partitioning into migrating and non-migrating parts of the boundary is shown in Fig. S5.

Stored energy The energy stored in the deformed microstructure can be estimated by summing up the energy of all the dislocation boundaries within the volume of interest. For this the dislocation boundary energy was determined using the standard approach based on the measured misorientation angle by application of the Read-Shockley equation [2]. In the present work a maximum energy of $\sigma_{max} = 0.324 J/m^2$ [3] for boundaries with misorientations higher than 15° in aluminum was used in a standard form of the Read-Shockley equation. To calculate the stored energy for each voxel in the deformed volume only dislocation boundaries to the first nearest-neighbor voxels are taken into account.

The local stored energy (as shown in Fig. 4a) at each voxel was estimated based on the kernel average misorientation (θ_{KAM}) of the voxel to its 26 nearest-neighbor voxels using the following equation:

$$E_s = 3Gb\ \theta_{KAM} / 2\Delta,$$

where G is the shear modulus, b is the Burgers vector, Δ is the step size. Further details about this method can be found in [4].

References:

1. Bachmann, F., Hielscher, R. & Schaeben, H. Texture analysis with MTEX – Free and open source software toolbox. *Solid State Phenomena* **160**, 63-68 (2010).
2. Read, W.T. & Shockley, W. Dislocation models of crystal grain boundaries. Phys. *Rev.* **78**, 275-289 (1950).
3. Murr, L.E. *Interfacial Phenomena in Metals and Alloys* (Addison-Wesley, Reading, MA, 1975).
4. Godfrey, A., Mishin, O.V. & Yu, T. Characterization and influence of deformation microstructure heterogeneity on recrystallization. *IOP Conf. Series: Mater. Sci. Eng.* **89**, 012003(2015).

Figures

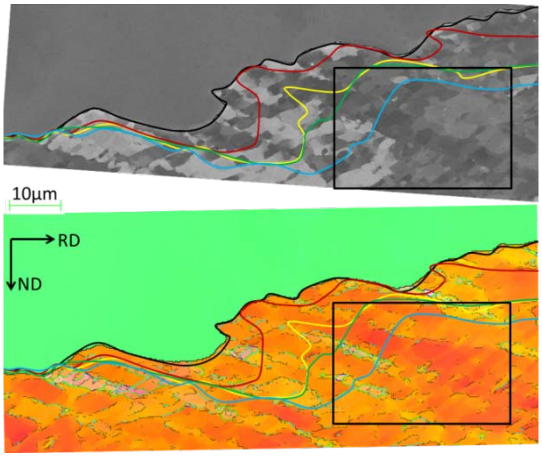

Fig. S1 2D ECC and EBSD characterization of the selected recrystallization boundary. Red, yellow, green and blue lines mark the boundary traces after preliminary annealing at 250°C for 15 min and 30 min, and at 260°C for 10 min and 30 min, respectively. The rectangle marks the area chosen for 3D mapping.

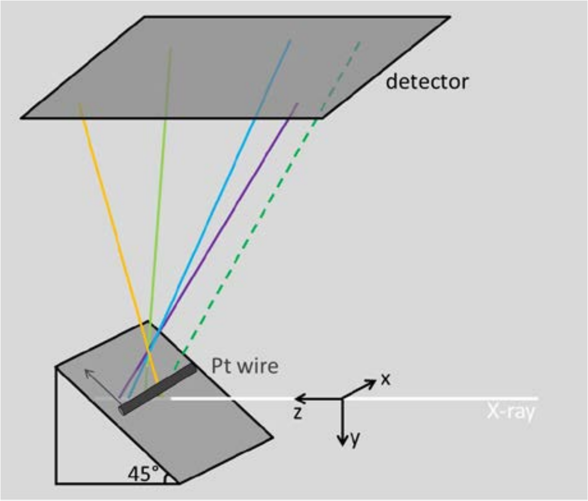

Fig. S2 A sketch showing the experimental setup of the X-ray Laue Diffraction Microscope.

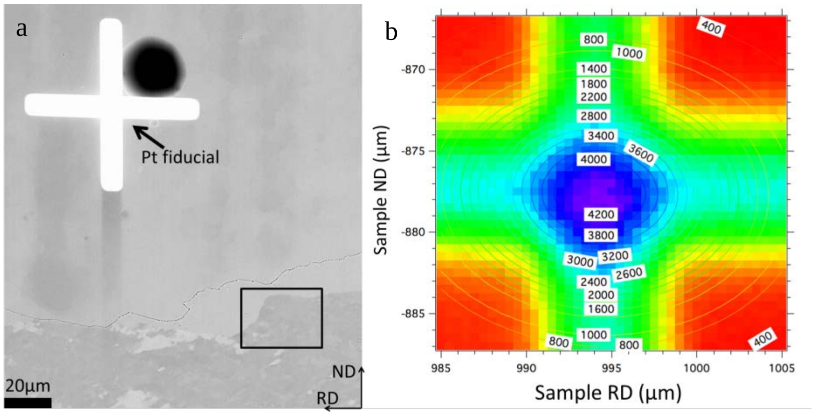

Fig. S3 Smaple alignment. (a) ECC image showing the Pt fiducial mark and its position relative to the mapping area (marked by the black rectangle). (b) Fluorescence scan of the Pt fiducial mark (units of fluorescence intensity). The peak position of the cross was fitted with a 2-D Gaussian function.

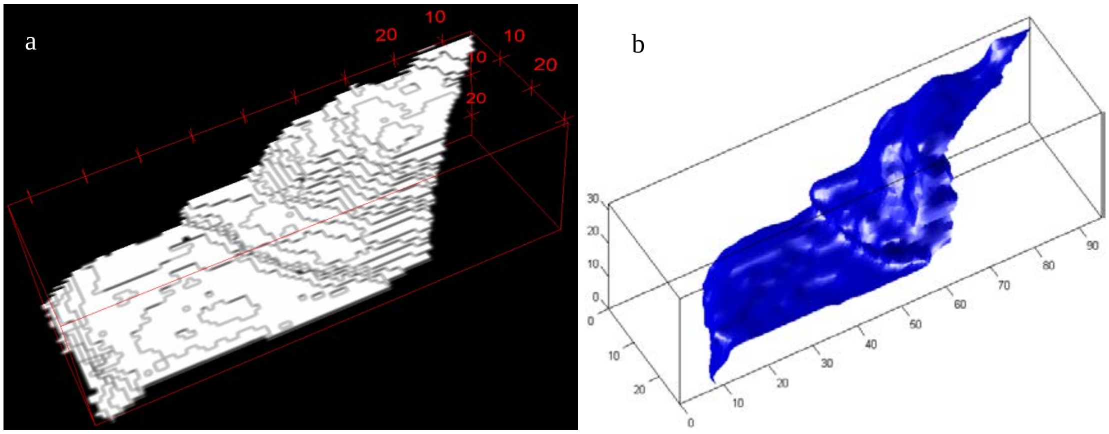

Fig. S4 Smoothening of the recrystallization boundary. The recrystallization boundary before annealing: (a) raw data and (b) after smoothing twice with 3x3 Gaussian filter.

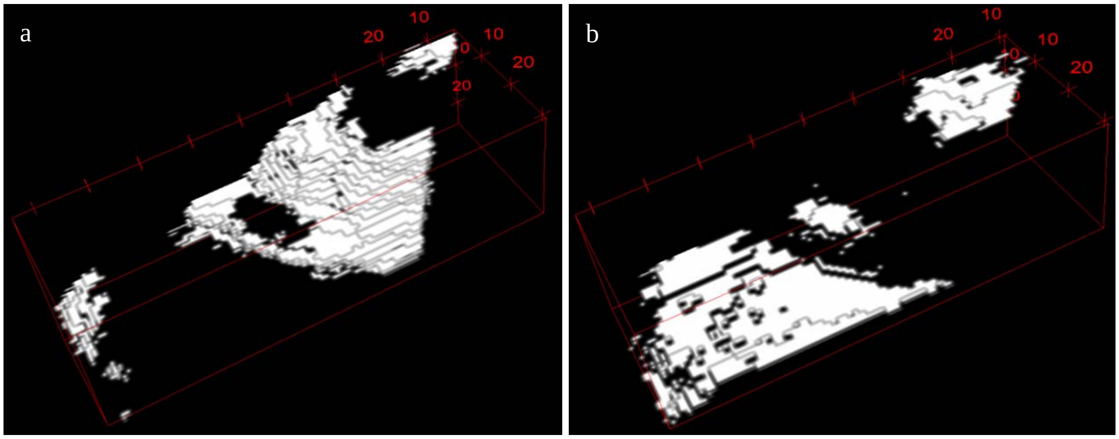

Fig. S5 Partitioning of the recrystallization boundary, based on the calculated migration distance at each position on the boundary, into (a) migrating parts and (b) non-migrating parts.
